# Microcosm biofilms cultured from different oral niches in periodontitis patients

**DOI:** 10.1080/20022727.2018.1551596

**Published:** 2018-11-27

**Authors:** Fabian Cieplik, Egija Zaura, Bernd W. Brandt, Mark J. Buijs, Wolfgang Buchalla, Wim Crielaard, Marja L. Laine, Dong Mei Deng, Rob A. M. Exterkate

**Affiliations:** a Department of Conservative Dentistry and Periodontology, University Medical Center Regensburg, Regensburg, Germany; b Department of Preventive Dentistry, Academic Centre for Dentistry Amsterdam (ACTA), University of Amsterdam and Vrije Universiteit Amsterdam, Amsterdam, The Netherlands; c Department of Periodontology, Academic Centre for Dentistry Amsterdam (ACTA), University of Amsterdam and Vrije Universiteit Amsterdam, Amsterdam, The Netherlands

**Keywords:** Biofilm, microcosm, subgingival, oral microbiome, 16S rDNA, periodontitis

## Abstract

**Objective**: Periodontal diseases are triggered by dysbiotic microbial biofilms. Therefore, it is essential to develop appropriate biofilm models. Aim of the present study was to culture microcosm biofilms inoculated from different niches in periodontitis patients and compare their microbial composition to those inoculated from subgingival plaque.

**Methods**: Saliva, subgingival plaque, tongue and tonsils were sampled in five periodontitis patients to serve as inocula for culturing biofilms *in vitro* in an active attachment model. Biofilms were grown for 14 or 28 d and analyzed for their microbial composition by 16S rDNA sequencing.

**Results**: As classified by HOMD, all biofilms were dominated by periodontitis-associated taxa, irrespective which niche had been used for inoculation. There was a low similarity between 14 d biofilms and their respective inocula (Bray-Curtis similarity 0.26), while biofilms cultured for 14 and 28 d shared high similarity (0.69). Principal components analysis showed much stronger clustering per patient than per niche indicating that the choice of patients may be more crucial than choice of the respective niches in these patients.

**Conclusion**: Saliva, tongue scrapings or tonsil swabs may represent sufficient alternative inocula for growing microcosm biofilms resembling periodontitis-associated microbial communities in cases when sampling subgingival plaque is not possible.

## Introduction

Periodontal diseases, including periodontitis and gingivitis, represent one of the most prevalent diseases in mankind, currently affecting about 538 million adults worldwide [1,2]. Accordingly, periodontitis is regarded to be the most common cause for tooth loss [3]. Periodontitis is a chronic inflammatory disease triggered by microbial biofilms on subgingival tooth surfaces and leads to the destruction of the periodontium, the tooth-supporting tissues [4]. According to the ‘ecological plaque hypothesis’, a certain stress (*e.g*. insufficient oral hygiene followed by accumulation of dental plaque) leads to an inflammatory host response and, as a consequence, to substantial environmental changes that eventually result in an ecological shift favouring the growth of proteolytic, anaerobic and Gram-negative bacterial species [5,6]. Recent concepts state that periodontitis is initiated by synergistic and dysbiotic microbiota, whose members (i.e. so-called commensals, accessory pathogens, inflammophilic pathobionts and keystone pathogens) fulfil distinct roles converging to disease-provoking microbiota [7,8]. Thereby, it seems to be less clear whether dysbiosis is causative or consequential of the disease [9].

As microbial interactions in such complex dysbiotic microbial communities may influence clinical treatment outcomes to a large extent [5,10,11], appropriate laboratory models mimicking subgingival and periodontitis-associated biofilms are of vital importance for investigating the efficacy of novel treatment modalities. In this light, many *in vitro* biofilm models have been developed, cultured from defined consortia comprising up to 15 representative bacterial and/or fungal species [12–16]. However, while these biofilms from defined consortia may be a worthy tool to study mechanistic aspects like inter-species interactions [12,17] or to investigate the mechanisms of action and damage patterns of given antimicrobials [16], they do not reflect the vast microbial complexity found in the oral cavity [18] and therefore are not suitable for studying biofilms at community-level [19]. An alternative to overcome this problem is to grow biofilms *in situ* on intra-oral appliances or splints that need to be carried by patients for given periods of time [20–22]. However, *in situ* studies, in general, are time-consuming and cost-intensive and one needs to count on the reliability of the participants to carry these uncomfortable appliances as specified by the investigators [19]. Furthermore, the number of biological replicates from a single participant may be limited as well. Yet another option combining advantageous features from both models described above is to take intra-oral microbiological samples from patients, *e.g*. plaque or saliva, and use these as *ex vivo* inocula to grow so-called microcosm biofilms *in vitro* [19,23–27]. These microcosm biofilms inoculated from patient samples are closer to the complex *in vivo* situation as compared to *in vitro* biofilms from defined consortia and exhibit easier handling and less dependence on participants as compared to biofilms grown *in situ* [19,24].

Recently, we could show that microcosm biofilms resembling subgingival periodontitis-associated biofilms can be cultured in a model using subgingival plaque sampled from periodontitis patients as an inoculum. This model supported the growth of fastidious anaerobic and proteolytic Gram-negative bacteria [25]. However, for practical reasons, a limited amount of subgingival plaque is available from a single patient. Consequently, culturing microcosm biofilms inoculated from samples taken from easier accessible niches than subgingival plaque would be valuable, especially for high-throughput evaluation of given antimicrobial or biofilm-modulating approaches, where usually higher sample amounts are needed for inoculation.

Therefore, the aim of the present study was to culture *in vitro* microcosm biofilms inoculated from different niches in periodontitis patients and compare their composition to those inoculated from subgingival plaque. For this purpose, saliva, subgingival plaque, tongue dorsum and tonsils were sampled from five patients suffering from chronic periodontitis and these samples were used as inocula for culture of microcosm biofilms.

## Materials and methods

### Patient selection

Patients were recruited from the patient pool of the Department of Periodontology, the Academic Centre for Dentistry Amsterdam (ACTA). For inclusion in this study, all patients had to have untreated chronic periodontitis showing at least one site with a pocket probing depth (PPD) of ≥6 mm in each quadrant, be >18 years old and have ≥20 teeth present. Patients were excluded if they had received periodontal treatment within the last three years, had used antibiotics within the last 6 months or used an antimicrobial mouth wash during the last 4 weeks. After detailed description of the study outline, written informed consent was obtained from all participants included in the study. The medical ethical approval for the protocol was obtained from the medical ethics committee of the Vrije Universiteit medical center (VUmc) Amsterdam (Amsterdam, the Netherlands; reference 2015.481).

In total, five patients were included (patient background and clinical data are shown in Table 1) from whom different niches (saliva, subgingival plaque, tongue dorsum and tonsils) of the oral cavity were sampled.

**Table 1.** Background and clinical data of the included periodontitis patients according to the intake.

Patient	Age	Gender	Ethnicity	Smoker	Medical history	N of teeth	Plaque [%]	N of teeth with PPD ≥6 mm	BOP [%]	Sampled sites	PPD in sampled sites [mm]
1	69	Female	Caucasian	Former*	Hypertension	23	52	7	47	16 mp	6
										26 mp	7
										35 mb	6
										46 mb	6
2	54	Male	Caucasian	Current	Hypertension	28	86	7	82	17 mp	8
										27 db	6
										36 ml	6
										46 dl	6
3	48	Female	Non-Caucasian	Never	Hypertension, diabetes	20	62	11	58	13 db	8
										27 mb	8
										35 dl	7
										41 l	7
4	76	Male	Caucasian	Never	Hypertension, hypercholesterolemia	23	48	9	50	14 dp	6
										24 dp	6
										34 mb	6
										43 mb	6
5	59	Male	Caucasian	Never	Hypertension	25	62	15	66	14 mb	8
										24 mb	6
										34 b	7
										47 b	10

*Former smoker = stopped smoking >1 year ago

### Sampling procedures and preparation of the samples

Prior to the sampling the patients were requested to follow specific instructions: the day before the appointment not to consume any alcohol; three hours before the appointment, a patient should not have brushed his/her teeth, have smoked or consumed anything except water. All sampling procedures were performed by one dentist (MLL) in the course of regular appointments of the patients at the Department of Periodontology (ACTA) according to a standardized procedure.

First, unstimulated saliva was collected using the spitting method [28]. In brief, patients were asked to let saliva gather on the bottom of their mouth and spit into a tube every 30 s for a total of 2 min. A total of 500 µL of saliva was transferred to a 1.5 mL Eppendorf tube containing 500 µL reduced transport fluid (RTF) [29]. For collecting subgingival samples, the deepest bleeding sites in each quadrant according to intake data were selected. Contamination with saliva was prevented by means of isolation with cotton rolls. Supragingival plaque was carefully removed and subgingival plaque was collected by inserting a sterile curette to the deepest point of the pocket and transferred to an Eppendorf tube containing 1 mL RTF. Tongue samples were collected in two distinct ways after isolation with tissues to prevent contamination by saliva: superficial layers of the tongue were sampled by performing five gentle zig-zag strokes from *papillae circumvallatae* to the anterior part of the tongue dorsum using a sterile microbrush (Microbrush International, Grafton, WI, USA). Then, the tip of the microbrush was cut and placed into an Eppendorf tube containing 1 mL RTF. The tongue dorsum was further sampled by performing one vigorous stroke with a tongue scraper (Meridol® Halitosis Tongue Cleaner, Therwil, Switzerland) from *papillae circumvallatae* to the anterior part of the tongue and the sampled biomass was transferred into an Eppendorf tube containing 1 mL RTF by means of a sterile spatula. Tonsil samples were obtained by performing a single stroke from both tonsils each using a sterile swab (Isohelix, Cell Projects, Harrietsham, UK). The swab was then transferred to an Eppendorf tube containing 1 mL RTF.

All Eppendorf tubes containing samples were centrifuged for 10 s at 6000 rpm (Microcentrifuge 3722L, Fisher Scientific International, Hampton, NH, USA), kept on ice and were further processed in the laboratory within 30 min. Samples were vortexed for 20 s and sonicated 20 s each (1 s pulsations at 40 W; Vibra cell, Sonics & Materials, Newtown, CT, USA) to separate aggregated bacteria. A total of 200 µL of each sample was transferred into a sterile Eppendorf tube and stored at −80° C for later processing for sequencing analysis of inoculum composition. The remaining 800 µL were used to inoculate biofilms.

### Inoculation and culture of biofilms

Biofilms were cultured in the so-called Amsterdam Active Attachment model (AAA-model), which is a high-throughput biofilm model based on active attachment of bacteria to different substrates and has been described in detail earlier [24]. In this study, the biofilm model consisted of a custom-made stainless steel lid with 24 clamps containing hydroxyapatite (HAP) discs (HIMED, Old Bethpage, NY, USA) that fitted on top of a 24-well polystyrene microtiter plate, allowing for 24 individual biofilms to form.

As biofilm culture medium, a basal liquid medium supplemented with proteose peptone was chosen, which was originally described by Thompson *et al*. [30]. Based on our previous study, this medium was modified by adding 30% of heat-inactivated foetal bovine serum (FBS; F4135, Sigma-Aldrich, St. Louis, MO, USA) [25].

As biofilms were cultured for two distinct total culture periods (14 and 28 days), two AAA-models were prepared for the samples obtained from one patient. Four subsamples were included per each of the sampled niches so that one AAA-model included all five inoculum types of a patient with four biofilms each.

For preparation of the inoculation media, the whole remaining sample (800 µL) from each niche was mixed with 14.3 mL biofilm culture medium (10.0 mL Thompson-medium and 4.3 mL FBS). Then, 1.5 mL was added per each well of a 24-well plate, and the models were subsequently incubated anaerobically (80% N_2_, 10% CO_2_, 10% H_2_) for 24 h for allowing initial attachment to the HAP discs. After this initial attachment period, the lids containing the HAP discs were transferred to 24-well plates containing fresh medium. During the further culture period of 2 or 4 weeks, respectively, medium was refreshed every 3.5 days.

### Harvesting of biofilms

Biofilms were harvested after total culture periods of 14 and 28 days. The HAP discs were carefully removed from the lids using sterile forceps and transferred to 2 mL of sterile phosphate-buffered saline (PBS). All samples were kept on ice. Biofilm dispersal was ensured by vortexing for 10 s, two sonication steps for 25 s each (1 s pulsations at 40 W; Vibra Cell, Sonics & Materials, Newtown, CT, USA) and vortexing again for 10 s. Complete biofilm removal from the HAP discs was confirmed visually. 1.4 mL of each dispersed biofilm was then transferred to a sterile Eppendorf tube and immediately frozen at −80° C for later processing for sequencing.

### Colony forming units assay

For estimating the number of total viable bacteria in the biofilms, a colony forming units (CFU) assay was employed. Harvested biofilms were tenfold serially diluted in cysteine peptone water (CPW) and were plated on Tryptic Soy Agar Blood plates. Plates were incubated for 7 d at 37°C under anaerobic conditions (80% N_2_, 10% CO_2_, 10% H_2_). Afterwards, total CFU were evaluated. CFU data was analyzed by means of SPSS, version 25 for Mac (SPSS Inc., Chicago, IL, USA) and medians, 1st and 3rd quartiles were calculated from the four replicates per patient, niche and culture period.

### DNA extraction, 16S rDNA sequencing and data analysis

Biofilm and inoculum samples were thawed and centrifuged at 13,000 rpm for 5 min at 4°C, supernatants were discarded and the pellet was resuspended in 100 µL sterile Tris-EDTA buffer. The resuspended samples were then added to wells of a 96-deep-well plate containing Tris-saturated phenol, 0.1 mm zirconium beads and lysis buffer. Samples were mechanically lysed by bead-beating at 1,200 rpm for 2 min and DNA was isolated with the Mag MiniKit (LGC Genomics, Berlin, Germany).

DNA concentrations were measured by means of qPCR as described earlier [31] and normalized to 2 ng per PCR reaction. The V4 region of the 16S rDNA gene (length ~252 nt) was amplified [32] with primers containing the respective Illumina adapters and a unique 8-nt index sequence key [33]. The amplification was performed according to Kozich *et al*. [33], except that 30 cycles were performed. The amplicons were pooled equimolarly and purified from agarose gel. Paired-end sequencing of the amplicons was conducted running the Illumina MiSeq reagent kit V3 (Illumina, Inc., San Diego, CA, USA) for 2 × 251 nt on the Illumina MiSeq platform at the VUmc Cancer Center Amsterdam (Amsterdam, the Netherlands). The flow cell was loaded with 12 pmol DNA containing 25% PhiX.

The obtained paired-end reads were merged, after which the resulting sequences (~252 nt long) were quality-filtered (max. expected error 0.5) and clustered into operational taxonomic units (OTUs) at 97% similarity as described previously [34]. However, a maximum of 25 mismatches (10%, as here the reads were 2 × 251 nt long) was used during the read merging step, before quality filtering. The most abundant sequence of each OTU was classified using the RDP classifier [35] (min. confidence 0.8) and the Human Oral Microbiome Database (HOMD; http://www.homd.org), version 14.51 [36].

The OTU table was subsampled at an equal depth of 1270 reads per sample to allow comparisons among the different samples. First, for assessing diversity within inocula and biofilms, the Shannon diversity index was calculated using PAST (PAlaeontological STatistics) version 3.20 [37]. Then, the data set was log_2_ transformed to normalize the data distribution for principal component analysis (PCA) by means of PAST and statistical significance was analyzed by one-way permutational multivariate analysis of variance (PERMANOVA) based on the Bray-Curtis similarity index. For pairwise comparisons, Bonferroni-corrected p-values were calculated (α = 0.05). Further, Bray-Curtis similarities between the samples were analyzed using SPSS, version 25 for Mac (SPSS Inc., Chicago, IL, USA).

## Results

### CFU assay

All biofilms showed growth of viable bacteria exhibiting median CFU-numbers ranging between 1.1 × 10^8^ and 3.7 × 10^9^ CFU per biofilm, irrespective of niche, patient or culture period.

### General sequencing output

The sequence data were clustered into a total number of 372 distinct OTUs (before subsampling). One inoculum (subgingival plaque from patient 4) yielded 14 reads only and was therefore excluded from further analysis. After subsampling (at an equal depth of 1,270 reads per sample), 317 OTUs remained. All OTUs lost due to subsampling had ≤10 reads in total.

The most abundant OTUs (taxonomic names of representative sequences of OTUs, based on HOMD) in this subsampled dataset were OTU 1 (*Parvimonas micra*; 54,745 reads), OTU 2 (*Peptostreptoccus stomatis*; 46,915 reads), OTU 3 (*Streptococcus anginosus*; 27,757 reads), OTU 4 (*Veillonella dispar*; 22,488 reads), OTU 6 (*Filifactor alocis*; 9,593 reads) and OTU 9 (*Prevotella intermedia*; 8,333 reads).

### Diversity

The median (min; max) number of OTUs was 56 (28; 106) in the inocula, 35 (20; 46) in biofilms grown for 14 days and 31 (20; 47) in biofilms grown for 28 days. The median (min; max) Shannon diversity index was 2.7 (1.8; 3.6) in the inocula, 2.2 (1.6; 2.7) in 14 d biofilms and 2.2 (1.7; 2.6) in 28 d biofilms. A detailed overview of the Shannon diversity values for all inocula and biofilms can be found in Supplementary Table 1.

### Reproducibility of the biofilms

For testing reproducibility of the biofilms, Bray-Curtis (BC) similarity was calculated between the four replicates of each biofilm. For biofilms cultured for 14 d, median (min; max) BC similarity was 0.80 (0.59; 0.88) between replicates, which declined to a median BC similarity of 0.78 (0.42; 0.88) for 28 d old biofilms.

### Similarity between inocula and biofilms

**Figure 1.**
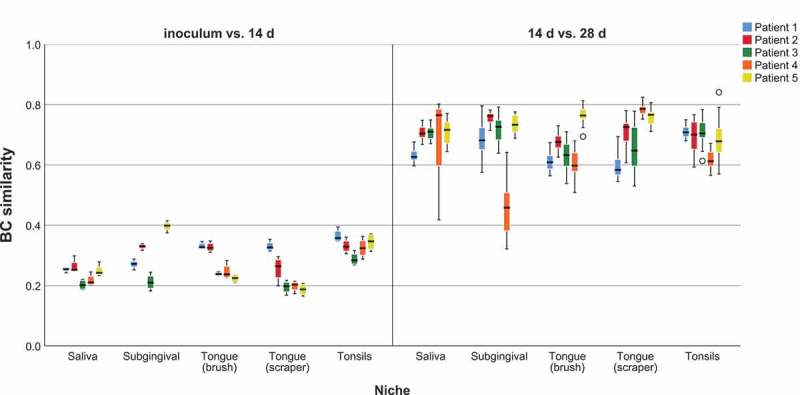
Bray-Curtis (BC) similarity between inocula and their corresponding biofilms. BC similarity between inocula and 14 d biofilms and between 14 d biofilms and 28 d biofilms shown per patient and per niche, respectively.

Similarity between the inocula and 14 d old biofilms as well as between 14 d and 28 d old biofilms was investigated using the Bray-Curtis (BC) similarity index and is depicted in Figure 1. While the median BC similarity between the inocula and biofilms cultured for 14 d (over all patients and niches) was 0.26 only, there was a high median BC similarity of 0.69 between biofilms cultured for 14 d and 28 d (over all patients and niches).

### Similarity between the subgingival inoculum and all other inocula and all biofilms

**Figure 2.**
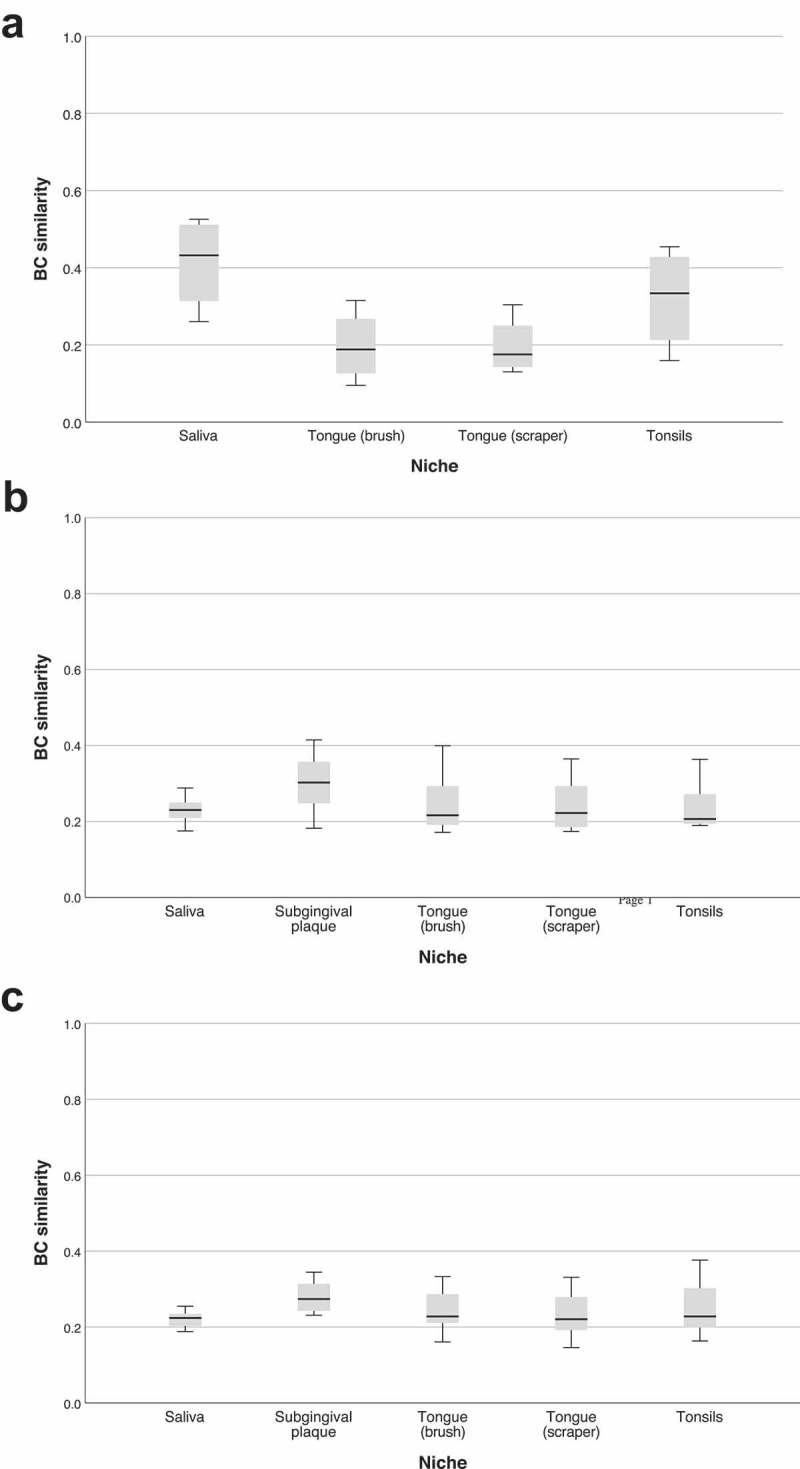
Bray-Curtis (BC) similarity between the subgingival inoculum and all other inocula and all biofilms (a). BC similarity between the subgingival inoculum and the other inocula. (b). BC similarity between the subgingival inoculum and 14 d biofilms. (c). BC similarity between the subgingival inoculum and 28 d biofilms.

Similarity between the subgingival inoculum and all other inocula as well as between the subgingival inoculum and biofilms cultured from all niches was investigated using the BC similarity index and is depicted in Figure 2. Regarding the inocula (Figure 2(a)), saliva exhibited the highest similarity to subgingival plaque (median 0.43), followed by tonsils (median 0.33). All biofilms showed low BC similarities between 0.21 and 0.30 (14 d) or between 0.22 and 0.27 (28 d) to the subgingival inoculum (Figure 2(b-c)).

BC similarities between biofilms cultured from the subgingival inoculum and biofilms from the other inocula showed higher median values around 0.5 (between 0.49 and 0.54 for 14 d biofilms and between 0.55 and 0.57 for 28 d biofilms; see Supplementary Figure 1).

### Taxonomy

To identify the most abundant OTUs that established in the biofilms at species level, the sequences were aligned to HOMD. In general, it could be observed that the differences between biofilms cultured for 14 d and 28 d were very small, which was also shown by BC similarity (see above, Figure 1). Likewise, differences between the niches were minor, in accordance to BC similarity (see above, Figure 2(b)).

Tables 2 and 3 show the OTUs that exhibited a relative abundance of ≥ 0.5% in the subgingival inocula or in the biofilms, respectively. While subgingival inocula comprised mostly of *Fusobacterium nucleatum ss. animalis* (OTU 15; 24.4% of total reads), *Streptococcusanginosus* (OTU 3; 5.6%) and *Alloprevotella tannerae* (OTU 50; 5.4%), biofilms were dominated by *Parvimonasmicra* (OTU 1; 21.5% of total reads), *Peptostreptococcus stomatis* (OTU 2; 18.5%), *Streptococcusanginosus* (OTU 3; 10.8%) and *Veillonelladispar* (OTU 4; 7.8%). Supplementary Tables 2 and 3 show most abundant OTUs for biofilms cultured from subgingival inocula and from saliva, respectively.

**Table 2.** OTUs exhibiting ≥0.5% abundance of reads in the subgingival inocula.

OTU	% reads	HOMD-based taxonomy of representative sequence	% confidence*
15	24.4	*Fusobacterium* *nucleatum ss. animalis*	100
3	5.6	*Streptococcus* *anginosus*	100
50	5.4	*Alloprevotella* *tannerae*	100
10	3.8	*Campylobacter rectus*/*showae*	98
71	3.3	*Bacteroidales sp*. oral taxon 274	100
5	3.1	*Streptococcus dentisani*/*infantis*/*mitis*/*oralis*/*sp*. oral taxon 058/*sp*. oral taxon 061/*sp*. oral taxon 064/*sp*. oral taxon 070/*sp*. oral taxon 423/*sp*. oral taxon 431/*tigurinus*	94
41	2.8	*Veillonellaceae*	100
68	2.6	*Treponema denticola*	99
251	2.6	*Leptotrichia*	100
1	2.0	*Parvimonas micra*	94
84	1.6	*Fretibacterium*	100
124	1.5	*Prevotella*	100
106	1.4	*Veillonella*	100
6	1.4	*Filifactor* *alocis*	100
35	1.3	*Anaeroglobus geminatus*	100
224	1.1	*Veillonella* *parvula*	84
67	1.1	*Tannerella forsythia*	99
148	1.0	*Treponema sp*. oral taxon 230	94
98	1.0	*Rothia dentocariosa*	100
188	0.9	*Tannerella sp*. oral taxon 286/*sp*. oral taxon 808	100
31	0.9	*Porphyromonas endodontalis*/*sp*. oral taxon 285	100
51	0.7	*Eubacterium brachy*	100
116	0.7	*Treponema lecithinolyticum*	97
13	0.7	*Eubacterium yurii ss. schtitka/yurii ss. yurii & margaretiae*	94
131	0.7	*TM7 sp*. oral taxon 346	86
151	0.7	*Leptotrichia sp*. oral taxon 498	100
227	0.7	*Eikenella corrodens*	98
120	0.7	*Bacteriodetes sp*. oral taxon 511	100
110	0.6	*Cardiobacterium valvarum*	100
54	0.6	*Leptrotrichia sp*. oral taxon 221	100
130	0.6	*Bacteriodetes sp*. oral taxon 507	100
79	0.6	*TM7 sp*. oral taxon 352	100
14	0.6	*Prevotella*	100
48	0.6	*Fretibacterium fastidosum*	100
74	0.6	*Corynebacterium matruchotii*	98
86	0.6	*Peptococcus sp*. oral taxon 167/*sp*. oral taxon 168	100
9	0.6	**Prevotella** *intermedia*	94
22	0.5	*Dialister* *invisus*	100
135	0.5	*Johnsonella ignava*	100
42	0.5	*Porphyromonas catoniae*/*sp*. oral taxon 275/*sp*. oral taxon 277/*sp*. oral taxon 284	100
165	0.5	*Prevotella fusca*	97
78	0.5	*Lachnoanaerobaculum*	100
81	0.5	*Prevotella oris*	100

*confidence of RDP classifier

**Table 3.** OTUs exhibiting ≥0.5% abundance of reads as average of all biofilms.

OTU	% reads	HOMD-based taxonomy of representative sequence	% confidence*
1	21.5	*Parvimonas micra*	94
2	18.5	*Peptostreptococcus* *stomatis*	98
3	10.8	*Streptococcus* *anginosus*	100
4	7.8	*Veillonella* *dispar*	91
6	3.7	*Filifactor* *alocis*	100
9	3.3	*Prevotella* *intermedia*	94
8	3.0	*Peptoniphilus* *lacrimalis*	100
7	2.7	*Fusobacterium* *necrophorum*	94
11	2.6	*Peptoniphilus sp*. oral taxon 386	100
15	1.6	*Fusobacterium* *nucleatum ss. animalis*	100
224	1.5	*Veillonella* *parvula*	84
19	1.5	*Mogibacterium diversum*/*neglectum*/*pumilum*/*vescum*	97
17	1.3	*Bacteroides heparinolyticus*	100
5	1.3	*Streptococcus dentisani*/*infantis*/*mitis*/*oralis*/*sp*. oral taxon 058/*sp*. oral taxon 061/*sp*. oral taxon 064/*sp*. oral taxon 070/*sp*. oral taxon 423/*sp*. oral taxon 431/*tigurinus*	94
24	1.2	*Eubacterium infirmum*	100
12	1.2	*Solobacterium moorei*	100
13	1.0	*Eubacterium yurii ss. schtitka*/*Eubacterium yurii ss. yurii* & *margaretiae*	94
286	1.0	*Parvimonas sp*. oral taxon 110	100
35	1.0	*Anaeroglobus geminatus*	100
106	0.9	*Veillonella*	100
22	0.8	*Dialister* *invisus*	100
23	0.8	*Bacteroidetes sp*. oral taxon 365	100
10	0.8	*Campylobacter rectus*/*showae*	98
20	0.7	*Dialister pneumosintes*	100
16	0.5	*Granulicatella adiacens*	97
28	0.5	*Porphyromonas gingivalis*	100

*confidence of RDP classifier

### Ordination of the biofilms by principal component analysis (PCA)

**Figure 3.**
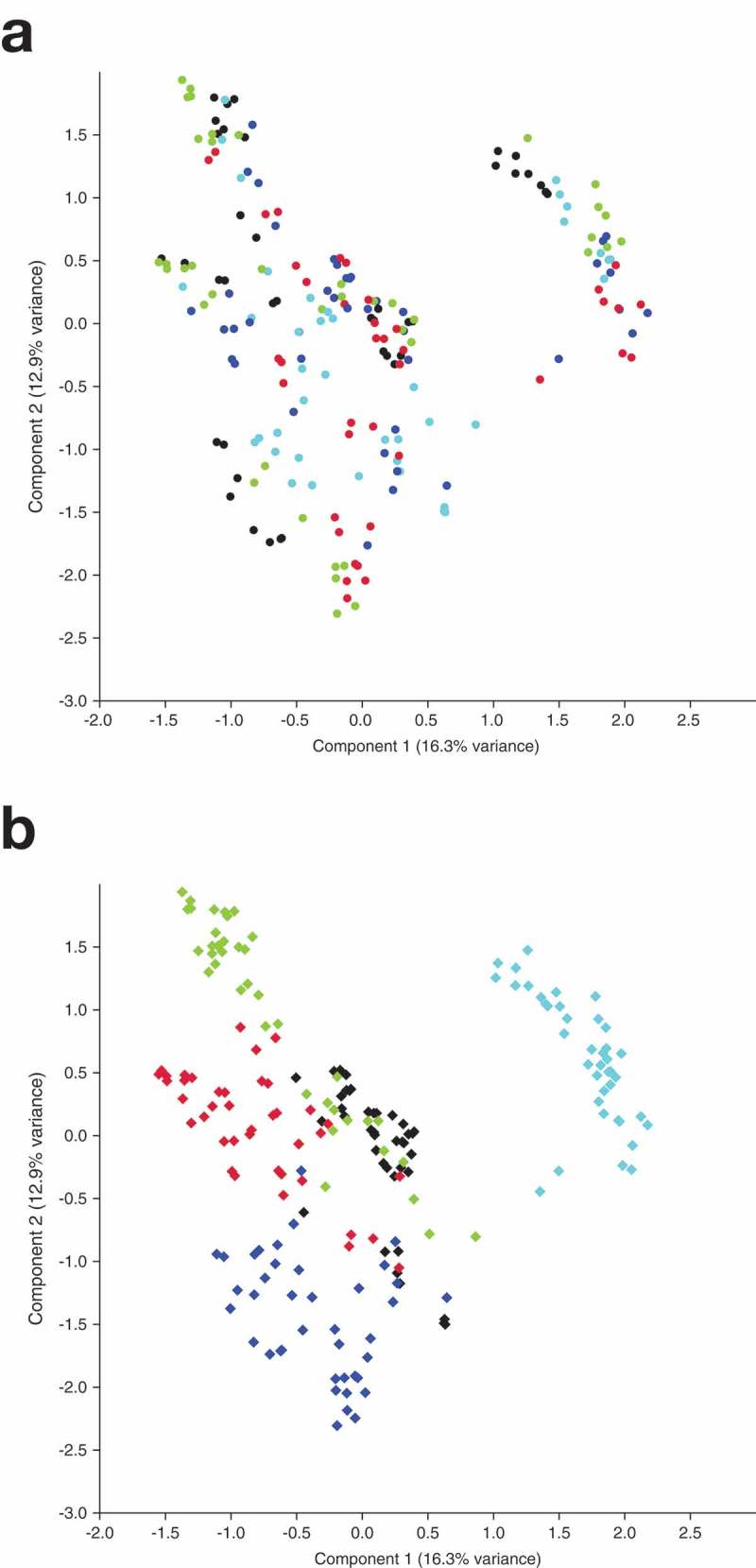
Principal component analysis (PCA) plots from biofilms. (a). PCA plot per niche. Each color represents a different niche, as follows: Black – Saliva, Aqua – Subgingival, Blue – Tongue (brush), Green – Tongue (scraper), Red – Tonsils. Differences between niches were statistically significant (PERMANOVA; p = 0.0001; *F *= 6.93). Pairwise comparisons showed significant differences between biofilms from all niches (p = 0.0001) except between biofilms grown from saliva and tongue (scraper) (p = 0.08) and biofilms grown from tongue (brush) and tongue (scraper) (p = 0.12). (b). PCA plot per patient. Each color represents a different patient, as follows: Black – Patient 1, Aqua – Patient 2, Blue – Patient 3, Green – Patient 4, Red – Patient 5. Differences between patients were statistically significant (PERMANOVA; p = 0.0001; *F *= 33.72) and pairwise comparisons showed significant differences as well (p = 0.0001 in all cases).

The microbiome data of the biofilms grown from all inocula were ordinated by applying principal component analysis (PCA) per niche (Figure 3(a)) and per patient (Figure 3(b)). Per niche, PERMANOVA revealed a significant difference between the niches (p = 0.0001; *F *= 6.93), while pairwise comparisons showed significant differences between biofilms from all niches (p = 0.0001) besides between the biofilms grown from saliva and tongue (scraper) (p = 0.08) and between the biofilms grown from the two tongue inocula (p = 0.12). In contrast, per patient there was a significant difference between all patients (p = 0.0001; *F *= 33.72) and by pairwise comparisons (p = 0.0001 in all cases).

In both PCA plots, the first component (x-axis) explained 16.3% of the variance among the samples and clearly separated Patient 2 from the other patients. Shifts on the x-axis to the left (patients 1, 3, 4 and 5) were mostly related to OTUs classified as *Prevotellaintermedia* (OTU 9; loading −0.36) and *Filifactoralocis* (OTU 6; −0.29), while shifts to the right (patient 2) were related to *Peptoniphilus lacrimalis* (OTU 8; 0.39) and to *Veillonella* sp. (OTU 106; 0.29) and *Veillonella parvula* (OTU 224; 0.24).

The second component (y-axis) explained a further 12.9% of the variance and displayed a clear separation between patients 3 and 4. Shifts on the y-axis to the upper direction (patient 4) were mostly related to the OTUs classified as *Fusobacterium necrophorum* (OTU 7; loading 0.3), *Dialister invisus* (OTU 22; 0.26) and *Oribacterium* sp. oral taxon 102 (OTU 40; 0.23), while shifts to the lower direction (patient 3) were related to *Filifactoralocis* (OTU 6; −0.33), *Bacteroides heparinolyticus* (OTU 17; −0.28), *Parvimonas* sp. oral taxon 110 (OTU 286; −0.25) and *Fusobacteriumnucleatum* subsp. *animalis* (OTU 15; −0.23).

Supplementary Figure 2 shows a PCA plot per patient including all biofilms and the subgingival inocula.

## Discussion

Periodontitis can usually be successfully treated by conventional non-surgical mechanical therapy, i.e. subgingival debridement, whereas some more aggressive and advanced cases may benefit from adjunctive therapies like administration of antibiotics to reach sufficient treatment outcomes [38,39]. However, in view of accelerating resistance rates among periodontal pathogens [40], the use of antibiotics in clinical periodontal practice is being increasingly criticized [41]. In this light, several ecology-based approaches like prebiotics or probiotics have been proposed [11,42–44]. For preclinical evaluation of these novel approaches, appropriate laboratory models are essential. Microcosm biofilms present simplified *in vitro* ecosystems that facilitate mimicking natural ecosystems under controlled conditions as well as monitoring microbial shifts upon given treatment modalities when combined with next-generation sequencing techniques [25]. In recent years, several microcosm biofilm models have been described [19,23–27] but – to the best knowledge of the authors – just two were developed with the rationale of periodontitis-associated subgingival biofilm models [25,27]. While in these previous studies, biofilms were inoculated from subgingival plaque sampled from periodontitis patients [25,27], the aim of the present study was to culture *in vitro* microcosm biofilms inoculated from other niches in periodontitis patients and compare their composition to those inoculated from subgingival plaque. In this instance, niches were selected based on easy accessibility and the possibility to reach higher sample amounts as compared to subgingival plaque, facilitating inoculation of larger numbers of biofilms and consequent high-throughput evaluation of given approaches. Therefore, saliva, tongue and tonsils were chosen, whereby tongue sampling was performed in two distinct ways.

For microcosm biofilms, the choice of growth conditions is a crucial factor, in particular when aiming for growth of fastidious anaerobes [23,25]. Based on our previous study [25], we employed a peptone-based medium, which has been originally described for culturing previously uncultured oral bacteria [30]. This growth medium comprises several amino acids, which are likely to be available in periodontal pockets due to proteolytic bacterial activity [45], as well as mucin, hemin and vitamin K, which all are known to support the growth of fastidious bacteria [30,46,47]. Serum was added to this medium to a concentration of 30%, as it constitutes the major component of gingival crevicular fluid (GCF) [48] and GCF in turn is continuously available in periodontal pockets as a nutrient source for bacteria in subgingival biofilms [5]. Furthermore, addition of serum to the growth medium led to an increase in biomass in the Zurich biofilm model [49]. HAP discs were used as biofilm substrate instead of glass discs used in the previous study [25] for the purpose of mimicking dental hard tissues and potentially enhancing attachment of the bacteria towards the substrate [50], which is a crucial point particularly in the active attachment model used in this study [24]. Two culture periods of 14 or 28 days, respectively, were chosen with the rationale of providing enough time for slow-growing bacteria to get established in the biofilms. Accordingly, Kistler *et al*. could show that there was a shift in microbial composition between 7 and 14 d in their biofilm model [26]. Growth medium was replenished every 3.5 days only as described earlier [25,26,30] to prevent over-growth of fast-growing species, *e.g*. streptococci.

All biofilms exhibited CFU-numbers between 10^8^ to 10^9^ within ≈ 1 log_10_ step indicating that there was no influence of any of the parameters (i.e. niche, patient, culture period) on the numbers of viable bacteria in the biofilms. Sequencing yielded between 28 and 106 OTUs for the inocula, wherefrom 20 to 47 OTUs were found in the microcosm biofilms. This was also reflected in the Shannon diversity index, whose median values slightly decreased from 2.7 in the inocula to 2.2 in the biofilm. However, this is still higher as compared to the previous study (inocula: 1.9–3.7; biofilms: 1.5–2.4), which may be explained by the fact that in contrast to the present study samples were not processed immediately then but first frozen and defrosted later for inoculation of the biofilms [25].

In our biofilms, there was a large difference in microbial composition between inocula and 14 d biofilms (BC similarity 0.26), while between 14 d and 28 d biofilms there was quite a high similarity (BC similarity 0.69). In addition, reproducibility between the four replicates of each biofilm was very high (BC similarity ~0.8). When comparing the four alternative inocula to the subgingival inoculum, saliva yielded the highest similarity (BC similarity 0.43) followed by tonsils (0.33). In contrast, all biofilms showed a low BC similarity with the subgingival inoculum (BC similarities between 0.21 and 0.3 for 14 d biofilms and between 0.2 and 0.27 for 28 d biofilms), irrespective of the niche used for inoculation. However, when looking at BC similarity between the biofilms cultured from the four alternative inocula and biofilms from the subgingival inoculum, there were high values between 0.5 and 0.6 (Supplementary Figure 1). Therefore, our growth medium apparently favoured growth of some OTUs and impeded it for others while shifting their microbial composition towards subgingival proteolytic biofilms and aligning all biofilms irrespective of the niche used for inoculation.

Accordingly, biofilms were dominated by periodontitis-associated taxa. *Parvimomas micra* (OTU 1), formerly referred to as *Peptostreptococcus micros*, was the most abundant OTU in the biofilms (21.5% of total reads) and has been strongly associated with periodontal disease [51,52]. Likewise, *Peptostreptococcusstomatis* (OTU 2; 18.5%) and *Filifactoralocis* (OTU 6; 3.7%), which have recently been unveiled as novel disease-associated species and key microbial players in periodontitis-associated dysbiosis [7,53], were highly abundant. The third-most abundant OTU *Streptococcusanginosus* (OTU 3; 10.5%) has also been suggested to reflect a diseased periodontal status [54]. Consequently, the microcosm biofilms can be considered to simulate periodontitis-associated microbial communities. Thereby, there were only slight taxonomic differences between saliva-derived biofilms and biofilms inoculated from subgingival plaque. Interestingly, the previous uncultured *Bacteroidetes sp*. oral taxon 365 (OTU 23) established at relatively high numbers in our microcosm biofilms (0.8% of total reads; 3.5% in saliva-derived biofilms). Nevertheless, several OTUs, which had originally been detected in the inocula, could not establish in the biofilms. As these ‘lost’ OTUs were from the same genera (*i.e. Neisseria, Leptotrichia, Rothia, Lachnospiraceae, Sneathia* and *Treponema*) as compared to the previous study, this may be a consequence of our choice of growth medium [25]. In this light, it may be interesting to evaluate other media in future studies, *e.g*. the so-called SHI medium, which has recently been developed by using DGGE profiling to combine the ingredients of several other media that supported growth of given subpopulations within the original microbial community [46]. Edlund *et al*. applied this medium for culture of microcosm biofilms from saliva and reported a high bacterial diversity in their biofilms (65 to 156 OTUs) [23]. However, it should be considered that biofilms were cultured only for 48 h because the goal of the study was just to mimic the original composition of the saliva samples [23].

Remarkably, PCA plots showed much stronger clustering of the biofilms per patient than per niche, indicating a strong patient-driven ‘fingerprint’. This is in line with the *F*-values from PERMANOVA, which were much higher per patient than per niche (*F *= 33.72 *vs. F *= 6.93) albeit p-values were strongly statistically significant (p = 0.0001 each). These results are in line with those from Rudney *et al*. who also found clear differences between subjects with regard to species composition in their microcosm biofilms [19]. Consequently, the choice of patients where samples are taken from may be more crucial for culture of microcosm biofilms than the choice of the respective niche in these patients. As different patients harbour different disease-associated microbial communities, they therefore may need different treatments to resolve their respective dysbiosis [10]. Consequently, it can be regarded to be a strong point of the model used in the present study that these differences between the patients can still be preserved even after 28 d of *in vitro* culture. This further allows for evaluation of novel treatment approaches on different disease-associated microbial communities *in vitro*, which is particularly essential for evaluation of novel ecology-based approaches for biofilm modulation, *e.g*. prebiotics or probiotics [42].

When developing *in vitro* biofilm models, critical choices need to be made. In the present study, five distinct oral niches in periodontitis patients were used as inocula for culturing microcosm biofilms that should resemble periodontitis-associated microbial communities. After 14 and 28 d of culture, biofilms from all niches had a relatively low similarity with subgingival plaque samples from the respective patients in terms of microbial composition, but – on the other hand – all biofilms were dominated by periodontitis-associated taxa. This was an effect of the chosen growth conditions, which favoured growth of proteolytic, anaerobic, Gram-negative bacteria. Therefore, the drawback of low similarity between the biofilms and the starting inoculum can also be seen as an advantage, meaning that microbiological samples from easier accessible niches than subgingival plaque can be as good inocula as subgingival plaque for culture of microcosm biofilms that resemble periodontitis-associated dysbiotic microbial communities.

Consequently, this study shows that saliva, tongue scrapings or tonsil swabs may be alternative inocula in cases when sampling subgingival plaque is not possible or when bigger volumes of microbiological samples from a single patient (or even multiple patients) are necessary. This may be especially worthwhile for high-throughput investigation of novel treatment approaches.
