# Supplementary material for: Microcosm biofilms cultured from different oral niches in periodontitis patients
Source: J Oral Microbiol. 2018 Nov 27;11(1):1551596. doi: 10.1080/20022727.2018.1551596 (PMC6263112; doi:10.1080/20022727.2018.1551596)
Supplement: Supplemental Material [file ZJOM_A_1551596_SM0078.docx]

**Microcosm biofilms cultured from different oral niches in periodontitis patients**

**– SUPPORTING INFORMATION –**

Fabian Cieplik^1,2,^*, Egija Zaura^2^, Bernd W. Brandt^2^, Mark J. Buijs^2^, Wolfgang Buchalla^1^, Wim Crielaard^2^, Marja L. Laine^3^, Dong Mei Deng^2^ and Rob A. M. Exterkate^2^

^1^ Department of Conservative Dentistry and Periodontology, University Medical Center Regensburg, Regensburg, Germany

^2^ Department of Preventive Dentistry, Academic Centre for Dentistry Amsterdam (ACTA), University of Amsterdam and Vrije Universiteit Amsterdam, Amsterdam, The Netherlands

^3^ Department of Periodontology, Academic Centre for Dentistry Amsterdam (ACTA), University of Amsterdam and Vrije Universiteit Amsterdam, Amsterdam, The Netherlands

* Corresponding author

Dr. Fabian Cieplik

Department of Conservative Dentistry and Periodontology

University Medical Center Regensburg

Franz-Josef-Strauß-Allee 11, 93053 Regensburg, Germany

email: [fabian.cieplik@ukr.de](mailto:fabian.cieplik@ukr.de)

**Supplementary Figure 1:** Bray-Curtis (BC) similarity between biofilms cultured from the subgingival inoculum and biofilms cultured from all other inocula

**Supplementary Figure 2:** Principal component analysis (PCA) plot from subgingival inocula and biofilms from all inocula.

Each color represents a different patient, as follows:

Black – Patient 1

Aqua – Patient 2

Blue – Patient 3

Green – Patient 4

Red – Patient 5

Each symbol represents a different timepoint, as follows:

X mark – subgingival inoculum

Circle – 14 d biofilms

Dot – 28 d biofilms

Differences between niches were statistically significant (PERMANOVA; p=0.0001; *F*=15.4). Pairwise comparisons were not possible as there was only one subgingival inoculum per patient.

**
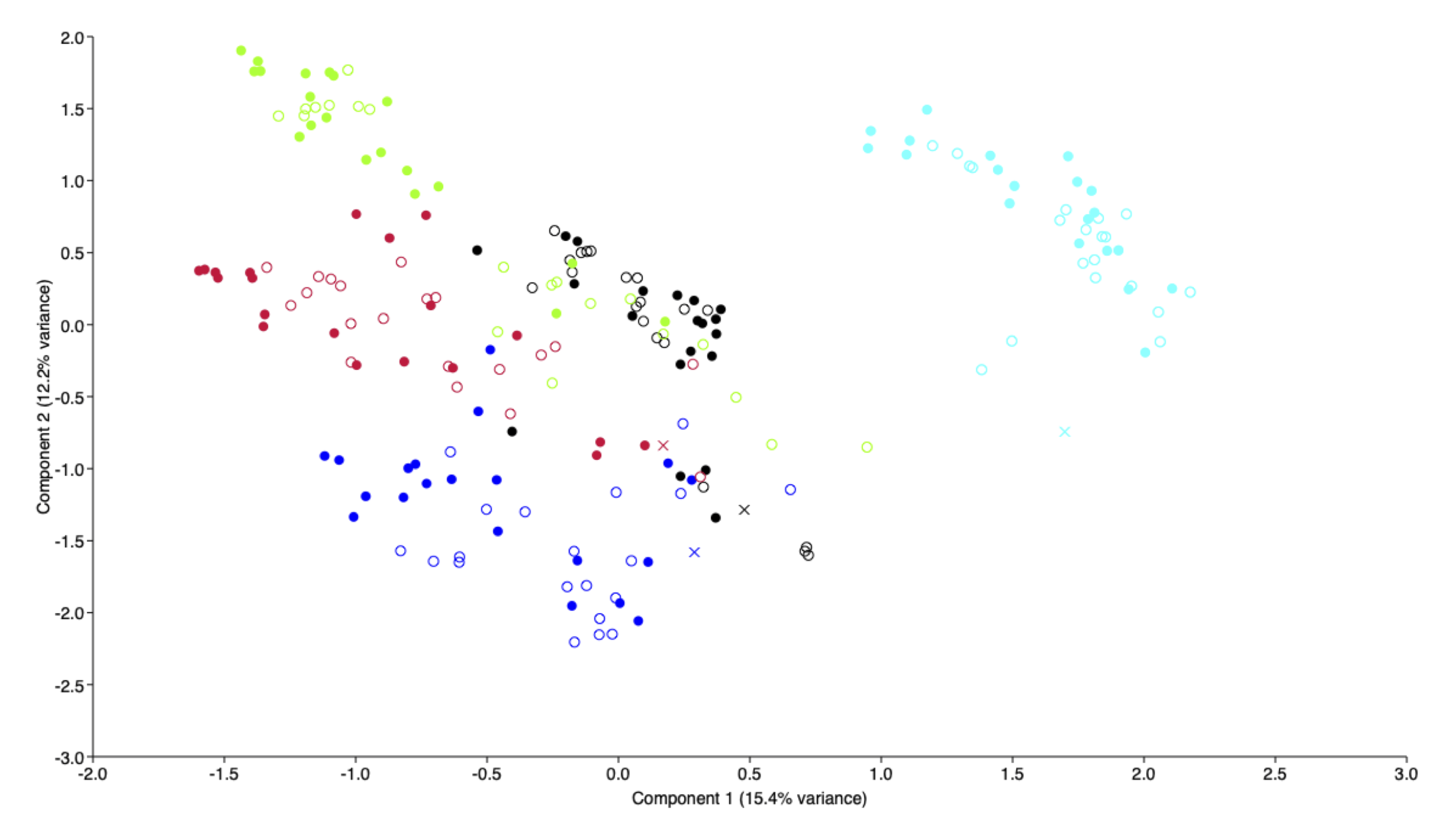
**

**Supplementary Table 1:** Shannon diversity index of all inocula and biofilms

| Patient | Niche | inocula | 14 d biofilms | 28 d biofilms |
| --- | --- | --- | --- | --- |
| Patient 1 | **Saliva** | 3.6 | 2.4 | 2.0 |
|  | **Subgingival plaque** | 3.0 | 2.0 | 1.8 |
|  | **Tongue (brush)** | 3.1 | 2.4 | 2.0 |
|  | **Tongue (scraper)** | 3.0 | 2.3 | 2.1 |
|  | **Tonsils** | 3.1 | 2.2 | 2.1 |
| Patient 2 | **Saliva** | 2.7 | 2.3 | 2.4 |
|  | **Subgingival plaque** | 3.3 | 2.2 | 2.2 |
|  | **Tongue (brush)** | 2.4 | 2.4 | 2.2 |
|  | **Tongue (scraper)** | 2.2 | 2.3 | 2.3 |
|  | **Tonsils** | 2.6 | 2.3 | 2.4 |
| Patient 3 | **Saliva** | 1.9 | 2.5 | 2.3 |
|  | **Subgingival plaque** | 3.1 | 2.1 | 2.0 |
|  | **Tongue (brush)** | 1.8 | 2.4 | 2.4 |
|  | **Tongue (scraper)** | 1.8 | 2.6 | 2.1 |
|  | **Tonsils** | 2.2 | 2.2 | 2.2 |
| Patient 4 | **Saliva** | 2.8 | 2.2 | 2.3 |
|  | **Subgingival plaque** | -* | 2.0 | 2.2 |
|  | **Tongue (brush)** | 2.6 | 2.2 | 2.1 |
|  | **Tongue (scraper)** | 2.6 | 2.3 | 2.3 |
|  | **Tonsils** | 2.8 | 2.0 | 2.1 |
| Patient 5 | **Saliva** | 2.5 | 2.1 | 2.4 |
|  | **Subgingival plaque** | 2.7 | 2.2 | 2.3 |
|  | **Tongue (brush)** | 2.4 | 2.1 | 2.2 |
|  | **Tongue (scraper)** | 2.4 | 2.2 | 2.3 |
|  | **Tonsils** | 3.0 | 2.0 | 2.1 |

* this inoculum showed 14 reads only and was therefore excluded for further analysis after subsampling

**Supplementary Table 2:** OTUs exhibiting ≥0.5% abundance of reads in the biofilms cultured from subgingival inocula

| OTU | % reads | HOMD-based taxonomy of representative sequence | % confidence* |
| --- | --- | --- | --- |
| 1 | 26.6 | *Parvimonas micra* | 94 |
| 3 | 13.8 | *Streptococcus anginosus* | 100 |
| 2 | 12.7 | *Peptostreptococcus stomatis* | 98 |
| 4 | 8.3 | *Veillonella dispar* | 91 |
| 6 | 4.2 | *Filifactor alocis* | 100 |
| 8 | 4.1 | *Peptoniphilus lacrimalis* | 100 |
| 35 | 3.5 | *Anaeroglobus geminatus* | 100 |
| 15 | 3.3 | *Fusobacterium nucleatum ss. animalis* | 100 |
| 9 | 3.0 | *Prevotella intermedia* | 94 |
| 13 | 1.6 | *Eubacterium yurii ss. schtitka* / *Eubacterium yurii ss. yurii* & *margaretiae* | 94 |
| 7 | 1.4 | *Fusobacterium necrophorum* | 94 |
| 20 | 1.1 | *Dialister pneumosintes* | 100 |
| 224 | 1.1 | *Veillonella parvula* | 84 |
| 12 | 1.0 | *Solobacterium moorei* | 100 |
| 27 | 1.0 | *Bacterioidaceae sp.* oral taxon 272 | 100 |
| 41 | 0.8 | *Veillonellaceae* | 100 |
| 106 | 0.8 | *Veillonella* | 100 |
| 22 | 0.8 | *Dialister invisus* | 100 |
| 18 | 0.7 | *Gemella haemolysans* / *morbillorum* / *sanguinis* | 100 |
| 26 | 0.7 | *Prevotella oralis* | 100 |
| 52 | 0.6 | *Prevotella baroniae* | 100 |
| 77 | 0.5 | *Capnocytophaga* | 100 |
| 19 | 0.5 | *Mogibacterium diversum* / *neglectum* / *pumilum* / *vescum* | 97 |
| 24 | 0.5 | *Eubacterium infirmum* | 100 |
| 61 | 0.5 | *Prevotella marshii* | 100 |
| 5 | 0.5 | *Streptococcus dentisani* / *infantis* / *mitis* / *oralis* / *sp.* oral taxon 058 / *sp.* oral taxon 061 / *sp.* oral taxon 064 / *sp.* oral taxon 070 / *sp*. oral taxon 423 / *sp.* oral taxon 431 / *tigurinus* | 94 |
| 16 | 0.5 | *Granulicatella adiacens* | 97 |

* confidence of RDP classifier

**Supplementary Table 3:** OTUs exhibiting ≥0.5% abundance of reads in the biofilms cultured from saliva inocula

| OTU | % reads | HOMD-based taxonomy of representative sequence | % confidence* |
| --- | --- | --- | --- |
| 1 | 21.4 | *Parvimonas micra* | 94 |
| 2 | 17.5 | *Peptostreptococcus stomatis* | 98 |
| 3 | 12.7 | *Streptococcus anginosus* | 100 |
| 11 | 6.4 | *Peptoniphilus sp.* oral taxon 386 | 100 |
| 4 | 4.9 | *Veillonella dispar* | 91 |
| 6 | 4.1 | *Filifactor alocis* | 100 |
| 7 | 3.8 | *Fusobacterium necrophorum* | 94 |
| 23 | 3.5 | *Bacteroidetes sp.* oral taxon 365 | 100 |
| 9 | 2.6 | *Prevotella intermedia* | 94 |
| 8 | 2.6 | *Peptoniphilus lacrimalis* | 100 |
| 19 | 1.9 | *Mogibacterium diversum* / *neglectum* / *pumilum* / *vescum* | 97 |
| 24 | 1.6 | *Eubacterium infirmum* | 100 |
| 13 | 1.4 | *Eubacterium yurii ss. schtitka* / *Eubacterium yurii ss. yurii* & *margaretiae* | 94 |
| 22 | 1.4 | *Dialister invisus* | 100 |
| 12 | 1.3 | *Solobacterium moorei* | 100 |
| 286 | 1.2 | *Parvimonas sp.* oral taxon 110 | 100 |
| 5 | 0.9 | *Streptococcus dentisani* / *infantis* / *mitis* / *oralis* / *sp.* oral taxon 058 / *sp.* oral taxon 061 / *sp.* oral taxon 064 / *sp.* oral taxon 070 / *sp*. oral taxon 423 / *sp.* oral taxon 431 / *tigurinus* | 94 |
| 45 | 0.9 | *Enterobacter* / *Klebsiella* / *Kluyvera* | 100 |
| 224 | 0.8 | *Veillonella parvula* | 0.84 |
| 26 | 0.8 | *Prevotella oralis* | 100 |
| 15 | 0.7 | *Fusobacterium nucleatum ss. animalis* | 100 |
| 17 | 0.7 | *Bacteroides heparinolyticus* | 100 |
| 33 | 0.6 | *Oribacterium sp.* oral taxon 102 | 100 |
| 20 | 0.5 | *Dialister pneumosintes* | 100 |
| 47 | 0.5 | *Olsenella sp. oral taxon 809* | 97 |

* confidence of RDP classifier
